# Probabilistic Circuit Implementation Based on P-Bits Using the Intrinsic Random Property of RRAM and P-Bit Multiplexing Strategy

**DOI:** 10.3390/mi13060924

**Published:** 2022-06-10

**Authors:** Yixuan Liu, Qiao Hu, Qiqiao Wu, Xuanzhi Liu, Yulin Zhao, Donglin Zhang, Zhongze Han, Jinhui Cheng, Qingting Ding, Yongkang Han, Bo Peng, Haijun Jiang, Xiaoyong Xue, Hangbing Lv, Jianguo Yang

**Affiliations:** 1 Zhejiang Lab, Hangzhou 311121, China; 20112020109@fudan.edu.cn (Y.L.); qhu@mail.ustc.edu.cn (Q.H.); hanyk@zhejianglab.com (Y.H.); pengb806@nenu.edu.cn (B.P.); jianghaijun@zhejianglab.com (H.J.); 2 School of Microelectronics, Fudan University, Shanghai 200433, China; xuexiaoyong@fudan.edu.cn; 3 School of Microelectronics, University of Science and Technology of China, Hefei 230026, China; wuqiqiao@mail.ustc.edu.cn (Q.W.); xuanzhi@mail.ustc.edu.cn (X.L.); chengjinhui@mail.ustc.edu.cn (J.C.); 4 Key Laboratory of Microelectronic Devices Integrated Technology, Institute of Microelectronics of Chinese Academy of Sciences, Beijing 100029, China; zhaoyulin@ime.ac.cn (Y.Z.); zhangdonglin20@mails.ucas.ac.cn (D.Z.); hanzhongze@ime.ac.cn (Z.H.); dingqingting@ime.ac.cn (Q.D.); lvhangbing@ime.ac.cn (H.L.)

**Keywords:** p-circuits, p-bits, invertible logic, TRNG based on RRAM, multiplexing strategy

## Abstract

Probabilistic computing is an emerging computational paradigm that uses probabilistic circuits to efficiently solve optimization problems such as invertible logic, where traditional digital computations are difficult to solve. This paper proposes a true random number generator (TRNG) based on resistive random-access memory (RRAM), which is combined with an activation function implemented by a piecewise linear function to form a standard p-bit cell, one of the most important parts of a p-circuit. A p-bit multiplexing strategy is also applied to reduce the number of p-bits and improve resource utilization. To verify the superiority of the proposed probabilistic circuit, we implement the invertible p-circuit on a field-programmable gate array (FPGA), including AND gates, full adders, multi-bit adders, and multipliers. The results of the FPGA implementation show that our approach can significantly save the consumption of hardware resources.

## 1. Introduction

In conventional computing systems, due to the constraints of the von Neumann architecture, huge computation and memory space and extremely long operation time are always required to solve many classical problems such as combinatorial optimization problems, neural networks, invertible logic, etc. To solve these problems effectively, new calculation methods have been continuously explored. Probabilistic computing, as a new computational paradigm, provides an attractive means to solve the above problem effectively with a small area and low power consumption, called a p-circuit [1,2]. The key role in the p-circuit is played by a probabilistic bit (p-bit), a robust unit fluctuating in time between 0 and 1, unlike conventional binary digital circuits where bits are used to represent a certain 0 or 1 [3], and a p-bit interacts with other p-bits in the same system using specific principles. Implementing the Boolean function with a p-circuit can achieve accuracy comparable to that of a standard digital circuit and, more importantly, is invertible, a unique feature not found in standard digital circuits. When operating in direct mode, the input is clamped, and the network provides the correct output. In the inverted mode, the output is clamped, and the network fluctuates between all possible inputs that are consistent with that output. When in floating mode, the network fluctuates between all the correct combinations of inputs and outputs.

Any random signal generator whose randomness can be tuned with a third terminal should be a suitable building block for the p-bit. In mathematical terms, p-bits can be described as Equation (1):

(1) $$\;m_{i}\left(t\right)=sgn\left\{rand\left(-1,1\right)+tanh\left(I_{i}\left(t\right)\right)\right\}$$

where rand (−1,1) represents a random number uniformly distributed between −1 and 1, tan h is an activation function, and sgn is the sign function (with binary 1 or −1 outputs). When the input $I_{i}\left(t\right)$ is zero, the output $m_{i}\left(t\right)$ takes on a value of −1 or 1 with equal probability. A large positive input makes the output more likely to be 1 while a large negative input makes the output more likely to be −1. Different p-bits can be interconnected according to

(2) $$I_{i}\left(t\right)=I_{0}\left(h_{i}+\underset{j}{\sum }J_{ij}m_{j}\left(t\right)\right)$$

where $J_{ij}$ is the coupling coefficient, $h_{i}$ is the external bias, and $I_{0}$ controls the strength of the interconnections. The values of $J_{ij}$, $h_{i}$ and $I_{0}$ are determined by the problem to be solved.

As mentioned above, one crucial part of constructing a p-circuit is implementing the p-bit in hardware. At present, there are three main ways to implement p-bits: Microcontrollers [3], digital circuits (field-programmable gate array (FPGA)/ASIC) [4,5], and new memory devices (MRAM, resistive random-access memory (RRAM)) [6,7,8]. There are two common problems in implementing p-bits with microcontrollers and digital circuits: One is the high energy consumption and the other is the waste of resources during operation. We can greatly reduce energy consumption by using new devices to implement p-bits, but as the number of p-bits increases, the variability between devices due to dispersion of the devices will need to be corrected [9]. We adopt the strategy of p-bit multiplexing, which reduces the waste of resources and avoids the additional design needed to correct the variability between devices, and finally achieves invertible logic with low power consumption and fewer resources.

## 2. Proposed P-Bit Design

**Figure 1:**
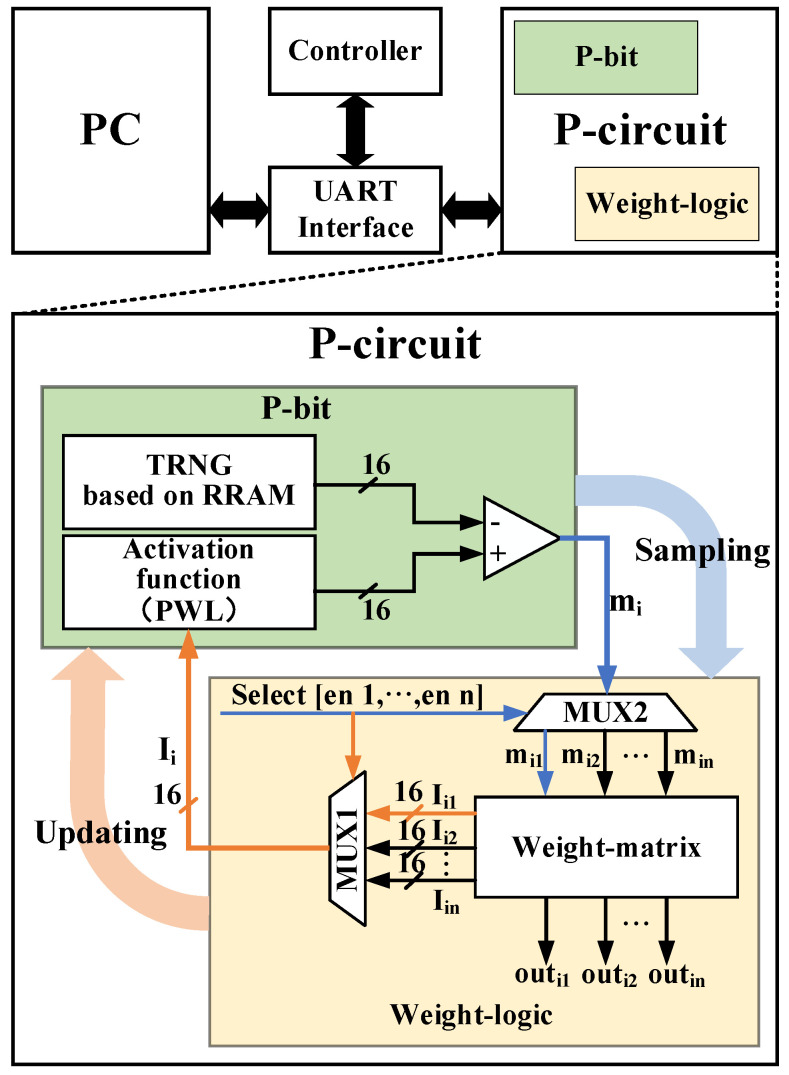
Block diagram of a probabilistic computing system including p-circuit, UART interface, PC, and controller (top). Detailed p-circuit structure (bottom): P-bit (green), weight-logic (yellow).

The overall structure diagram of the proposed probabilistic computing system is shown in Figure 1, which mainly includes the p-circuit, UART (Universal Asynchronous Receiver/Transmitter) interface, PC, and controller. The p-circuit completes the invertible logic, and the UART interface collects data and transmits it to the PC, the most important part of which is the components and implementation of the p-circuit. The p-circuit is the key to implementing invertible logic, which consists of a p-bit and weight-logic. In this section and the next section, we will describe and show the realization of the p-bit and weight logic in detail, respectively.

Since signals are represented by definite 0 and 1 in digital circuits, we convert Equation (1) into the following form:

(3) $$\;m_{i}\left(t\right)=step\left\{rand\left(0,1\right)-sigmoid\left(I_{i}\left(t\right)\right)\right\}$$

where rand(0,1) can be generated by the RRAM-based true random number generator (TRNG), the sigmoid function can be implemented by piecewise linear approximation (PWL), and the step function can be completed by the comparator. We will describe the specific implementation of these three parts separately below.

### 2.1. TRNG Based on RRAM

**Figure 2:**
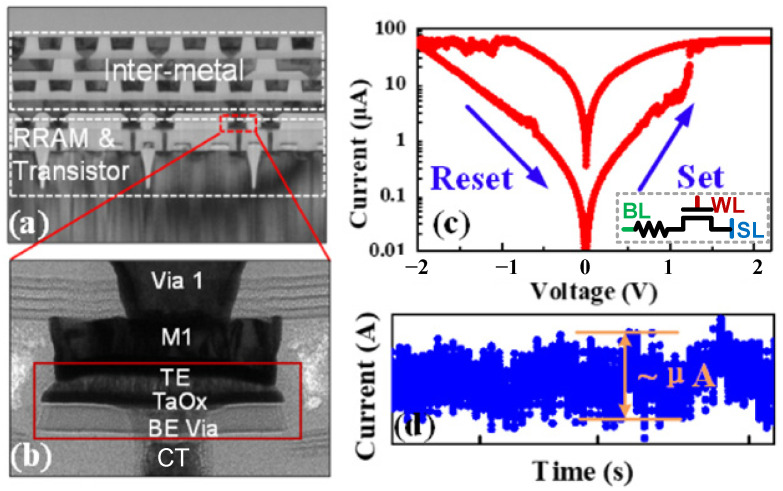
Structure and characteristics of RRAM. (**a**) The cross-section of RRAM cell in a 28 nm process 1T1R array. The RRAM unit is built between contact and Metal 1; (**b**) the magnified view of the cell structure; (**c**) typical DC I-V characteristics of the RRAM cell and structure of 1T1R RRAM; (**d**) the noise measured in the 28 nm RRAM cell; the array size is 16 Kb.

Compared with the traditional CMOS random number generator, the random number generator designed with RRAM has the advantages of a small area, low power consumption, and good randomness, which is more attractive [10,11,12,13]. The RRAM stack includes the bottom electrode (BE, TiN), top electrode (TE, TiN), and TaOx layer. The TaOx is deposited by PVD serving as the switching layer. Figure 2a shows the cross-section of RRAM cells in the array used in this study. The RRAM is built between contact (CT) and Metal 1 (M1). The magnified view of the RRAM cell structure is shown in Figure 2b. A TaOx switching layer was formed in the trench of M1 in connection with CT. An interface layer with a thickness of 1.5 nm∼5 nm was formed between the TaOx layer and TE. The detailed process flow of the RRAM device can be found in our previous work [14]. Figure 2c illustrates the typical DC I-V characteristics of the RRAM cell measured at room temperature, demonstrating a low 80 µA switching current and a low operation voltage below 1.5 V. During the DC test, the bias voltage of the transistor (NMOS) is 1.4 V due to the need for current limiting protection of the RRAM device during the set operation, while the SL is grounded, and the BL provides different DC voltages. For reset operation, the transistor bias voltage is 2.5 V, while BL is grounded, and SL provides different DC voltages.

**Figure 3:**
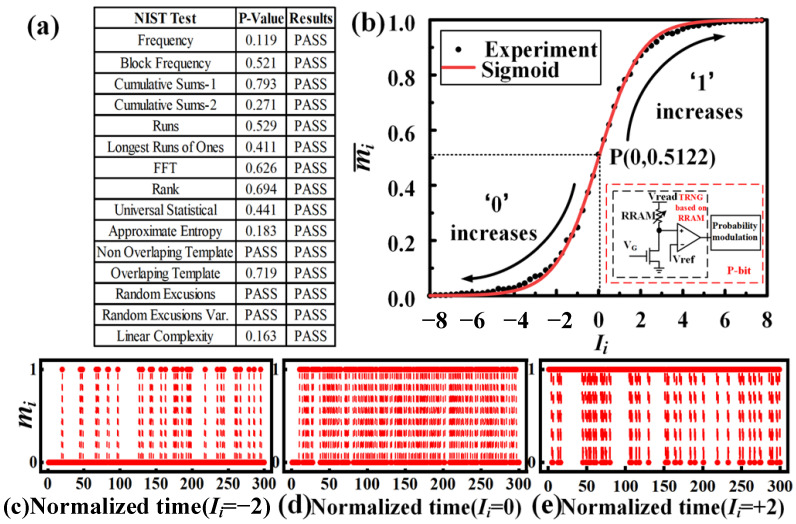
Probabilistic-adjustable randomness of P-bit. (**a**) NIST-verified results at p = 0.5; (**b**) the time-averaged output of p-bit, which fits sigmoid well; (**c**–**e**) the statistical value of $m_{i}$ when $I_{i}$ is equal to −2, 0, +2, respectively, over 300 cycles.

The noise (including RTN [15] and flicker noise) affected by the local trap (a crystal defect or chemical center in a semiconductor capable of capturing electrons or holes) is shown in Figure 2d. The noise is utilized as the entropy source for the p-bit circuit in this work. The amplitude of the noise in the 28 nm RRAM can reach the order of microamps, making it easier to detect and utilize the noise to form a true random number generator compared to the noise of other new devices [16]. The noise extraction circuit is shown in the inset in Figure 3b. When the $V_{G}$ is high, the transistor is turned on, the corresponding RRAM unit is selected, and a small read voltage ($V_{read}\;$= 0.3 V) is applied to the RRAM. Due to the internal noise of the RRAM, the voltage at the positive input of the comparator is not fixed but has small voltage fluctuations. Upon comparing the $V_{ref}$ with it, the comparator will output random 0 and 1 results. NIST 800-22 (National Institute of Standards and Technology, Gaithersburg, MD, USA) randomness test suites are performed on 10 M bits collected from the RRAM chip. As shown in Figure 3a, the data passed all test items, showing a high quality of randomness. The generated true random number will be used by the probability modulation module including the sigmoid function circuit and comparator to realize the bitstream with adjustable probability. Figure 3b shows the average value of $m_{i}$ obtained after 10^6^ cycles of sampling when the input changes, which highly fits the sigmoid function. Figure 3c–e shows the statistical value of $m_{i}$ when $I_{i}$ is equal to −2, 0 and +2, respectively, over 300 cycles. When performing FPGA function implementation, we first sample and collect the random number sequence generated by the RRAM-based TRNG and store it in RAM, which continuously outputs the random number sequence to verify the function of the circuit.

### 2.2. Sigmoid Function

We use PWL [17] to implement the sigmoid function in the digital method, which is different from [4] using a lookup table (LUT) and [5] using a finite state machine (FSM). Since the input–output curve of the P-bit is highly coincident with the sigmoid function, and the sigmoid function tends to saturate when the input exceeds the interval [−8, +8], we restrict the input range of the sigmoid function to the interval [−8, +8] and use fifteen broken lines to approximate the sigmoid function. Then, the input and output of the sigmoid function are quantized by a 16-bit fixed-point number. For the input, we use one MSB to represent the sign, the middle three bits to represent the integer, and the rest to represent the decimal. For the output, all 16 bits are used to represent the decimal. Table 1 shows the truth table of the 15-segment polyline approximating the sigmoid function for x > 0 only, due to the symmetry of the sigmoid function. Taking the input range of 0 to 1 as an example, the slope of the broken line is 0.25, so the output starts at 0.5, and the decimal part of the input is shifted to the right by 2 places to achieve multiplication by 0.25. In this way, the use of multipliers can be avoided, effectively saving hardware resources.

### 2.3. Comparator

A 16-bit comparator compares the outputs of the sigmoid function and the TRNG. If the output of the activation function is larger than TRNG, the output $m_{i}$ is 1; otherwise, the output is 0. Figure 3b shows the time-average characteristics of p-bit, where $\bar{m_{i}}$ is the statistical average value of $m_{i}$ over 10^6^ sampling cycles. When the input $I_{i}$ is 0, $m_{i}$ randomly fluctuates between 0 and 1 with equal probability, so the time-average output $m_{i}$ is approximately equal to 0.5. As the input $I_{i}$ increases, the number of 1 generated by the p-bit will exceed the number of 0, increasing the time-averaged output $\bar{m_{i}}$.

## 3. Weight-Logic Implementation

As mentioned above, the p-circuit consists of a p-bit and weight-logic. The p-bit is presented in Section 2, and adjusts the probability of output 1 according to the calculation result of the weight-logic and waits for the weight-logic to sample. In this section, we will show the design of the weight-logic, including the calculation of the weight-matrix and the multiplexing strategy applied therein.

### 3.1. Weight-Matrix

**Figure 4:**
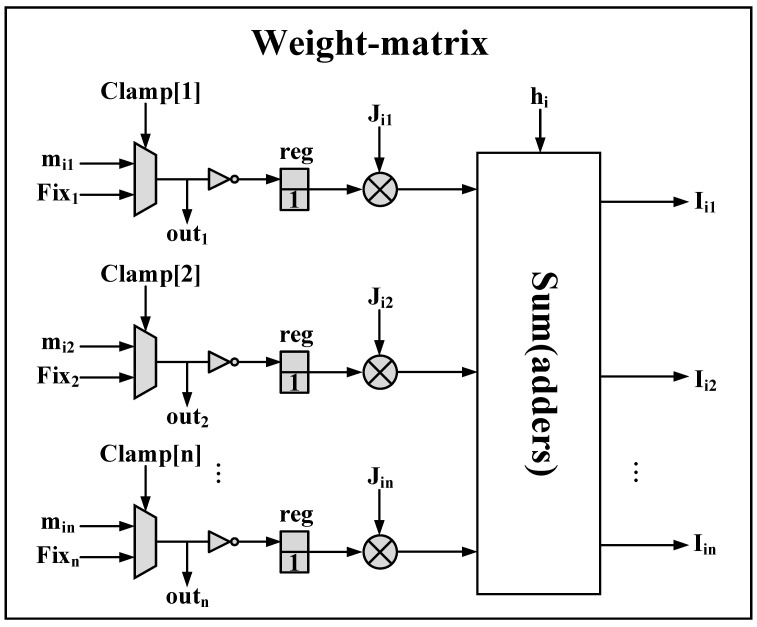
The structure of the weight-matrix. MUXs are used to choose the fluctuation value ($m_{in}$) or a certain value ($Fix_{n}$). 2-bit signed registers with LSB fixed to 1 are used to convert p-bit output m∈{0,1} to a bipolar m∈{−1,+1} representation.

The weight-matrix implements Equation (2) employing adders and multipliers, the structure of which is shown in Figure 4. Sometimes it is necessary to fix the output of some p-bits to a certain value, so we use MUXs to choose between the fluctuation value ($m_{in}$) and the certain value $(Fix_{n}$), which is controlled by the Clamp[1:n]. Since $m_{j}$ in Equation (2) is bipolar ($m_{j}\in \left\{-1,+1\right\}$), the p-bit output $m_{i}$ in Equation (3) is unipolar ($m_{j}\in \left\{0,1\right\}$), so we use 2-bit signed registers with LSB fixed to 1 for the conversion instead of linear mapping through y=2×m−1, which can reduce one multiplication and one additional operation.

### 3.2. Multiplexing Strategy

**Figure 5:**
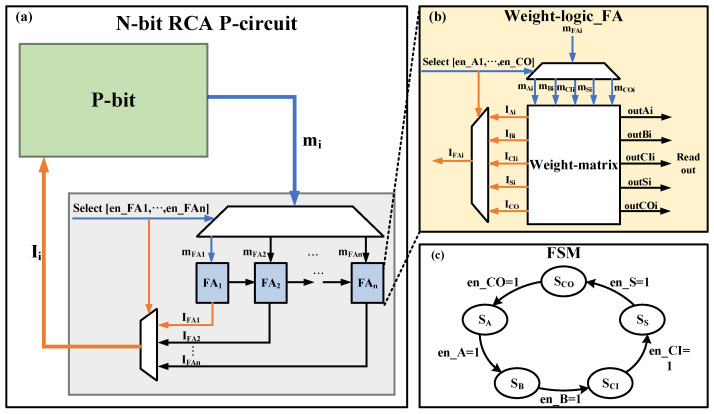
Proposed N-bit ripple carry adder (RCA). (**a**) The structure of N-bit RCA p-circuit using two-multiplexing strategy; (**b**) the structure of FA using time-division multiplexing; (**c**) the calculation process in the weight-matrix.

For every p-circuit, there is a requirement that the p-bit must update serially. In [4], an additional sequencer circuit is used to force an updating sequence between p-bits. In this paper, we propose a multiplexing strategy to reduce the number of p-bits and complete the serial update of p-bits through FSM in the weight-matrix. Taking the N-bit ripple carry adder (RCA) as an example, in which we adopt the two multiplexing strategies. The first multiplexing strategy is applied to the basic unit full adder (FA) of RCA as shown in Figure 5b,c. Usually, it takes five p-bits to construct an FA, where *A*, *B*, and *CI* are the inputs of the FA, and *S* and *CO* are the outputs of the FA. In this work, we only need one p-bit as a generator to produce 0 or 1 probabilistically, while some registers store the states of the p-bit. This multiplexing strategy not only performs serial updates naturally but also greatly reduces the number of p-bits. We use two MUXs to achieve p-bit time-division multiplexing as shown in Figure 5b. The signal to control the MUX is generated by the weight-matrix module, and the concrete operation process is shown in Figure 5c. Starting with state 1, the circuit accomplishes two things in each state: Firstly, the input of the corresponding p-bit is calculated based on the interconnect coefficient ($J_{ij}$), the external bias ($h_{i}$), and the p-bit output ($m_{i}$; secondly, the corresponding control signal is set to 1 to update the p-bit. An FA needs to go through five states to complete an update of all bits. The second multiplexing strategy is applied to N-bit RCA as shown in Figure 5a, and the update order is from FA_1_ to FA_n_. Although the multiplexing strategy increases the operation time, it is acceptable for statistical-based probabilistic computing to reduce hardware consumption.

## 4. Implementation Results

This section shows simulation results of the invertible AND gate, FA, 16-bit RCA, and 4-bit multiplier. The implementation of the invertible circuits in this section is based on the mathematical description of the p-bit and the coupling relationship between p-bits, as shown in Equations (1) and (2). In hardware, they correspond to the p-bit module and weight-logic module, respectively. The coupling weights $J_{ij}$ between p-bits are determined by the invertible logic problem to be implemented. The p-circuits system constantly updates its values and obtains the correct solution to the problem with a higher probability.

### 4.1. Invertible AND Gate and Full Adder (FA)

The invertible AND gate and FA can be implemented as p-circuits following the architecture of Figure 1 using the matrix $J_{AND}$, $J_{FA}$ and vector $h_{AND}$, $h_{FA}$ in Equations (4) and (5) [18].

(4) $$\;J_{AND}=\left[\begin{array}{ccc} 0 & -1 & +2\\ -1 & 0 & +2\\ +2 & +2 & 0 \end{array}\right]\;h_{AND}=\left[\begin{array}{c} +1\\ +1\\ -2 \end{array}\right]$$

(5) $$J_{FA}=\left[\begin{array}{ccc} \begin{array}{ccc} 0 & -1 & -1\\ -1 & 0 & -1 \end{array} & \begin{array}{c} +1\\ +1 \end{array} & \begin{array}{c} +2\\ +2 \end{array}\\ \begin{array}{ccc} -1 & -1 & 0\\ +1 & +1 & +1 \end{array} & \begin{array}{c} +1\\ 0 \end{array} & \begin{array}{c} +2\\ -2 \end{array}\\ \begin{array}{ccc} +2 & +2 & +2 \end{array} & -2 & 0 \end{array}\right]\;h_{FA}=\left[\begin{array}{c} 0\\ 0\\ \begin{array}{c} 0\\ \begin{array}{c} 0\\ 0 \end{array} \end{array} \end{array}\right]$$

**Figure 6:**
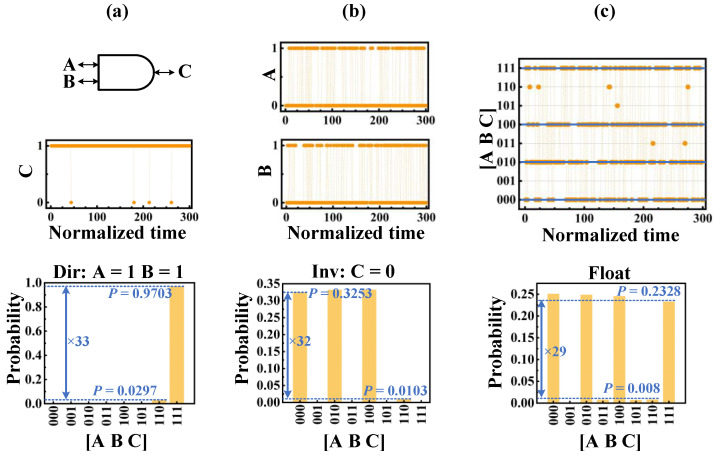
Invertible AND gate operation. (**a**) Directional mode: Clamping the inputs (*A*, *B*) to (1,1), the time-dependent output of *C* for the AND gate (top), the statistics collected for 10^6^ samples (bottom); (**b**) inverse mode: Clamping the outputs *C* to 0, time-dependent outputs of (*A*, *B*) for the AND gate (top), the statistics collected for 10^6^ samples (bottom); (**c**) floating mode: All the inputs and output are floating, time-dependent nodes of (*A*, *B*, *C*) for the AND gate (top), the statistics collected for 10^6^ samples (bottom).

**Figure 7:**
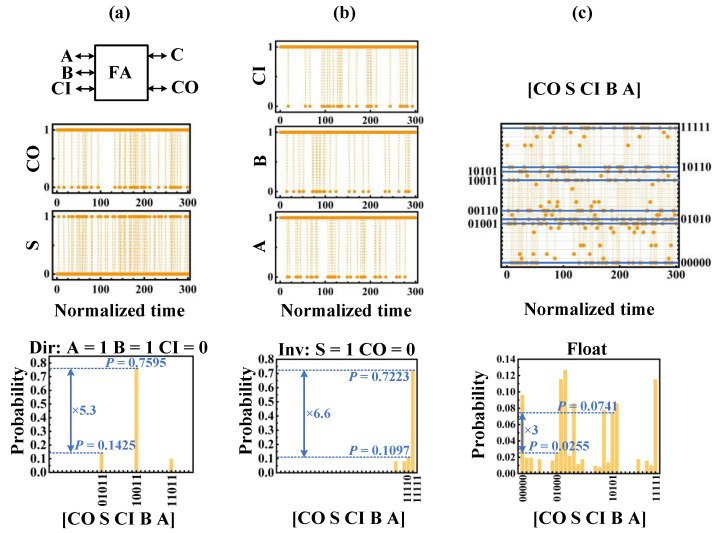
Invertible full adder operation, similar to invertible AND gate including directional mode (**a**), inverse mode (**b**), and floating mode (**c**).

For the invertible AND gate and FA, we have implemented three different modes: The directional mode, which clamps the inputs, the inverse mode, which clamps the output, and the floating mode where the input and output are floating. Figure 6 and Figure 7 show the steady-state statistics of the AND gate and FA. In Figure 6a, inputs *A* and *B* are clamped at 1 by *Clamp* and *Fix_n_* signals, and output *C* is held at 1 for a long time during 300 sampling cycles. Figure 6b shows the inverse mode where output *C* is clamped to 0. From the figure, the three combinations of input (*A, B*) have a relatively higher probability of occurrence, namely (0,0), (0,1), and (1,0), which is consistent with the truth table of the AND gate. In Figure 6c, all the inputs and output are floating, and the probabilities of all correct AND gate input and output combinations are significantly high, close to 0.25. Similar to the invertible AND gate, Figure 7 shows the results of the invertible FA. FA works in the directional mode when the input (*A*, *B*, *CI*) is clamped to (1,1,0), in the inverse mode when the output (*S*, *CO*) is clamped to (1,1), and in the floating mode when the input and output are floating. The invertible AND gate and FA require three clock cycles and five clock cycles, respectively, to complete a calculation.

### 4.2. 16-Bit Ripple Carry Adders (RCA)

**Figure 8:**
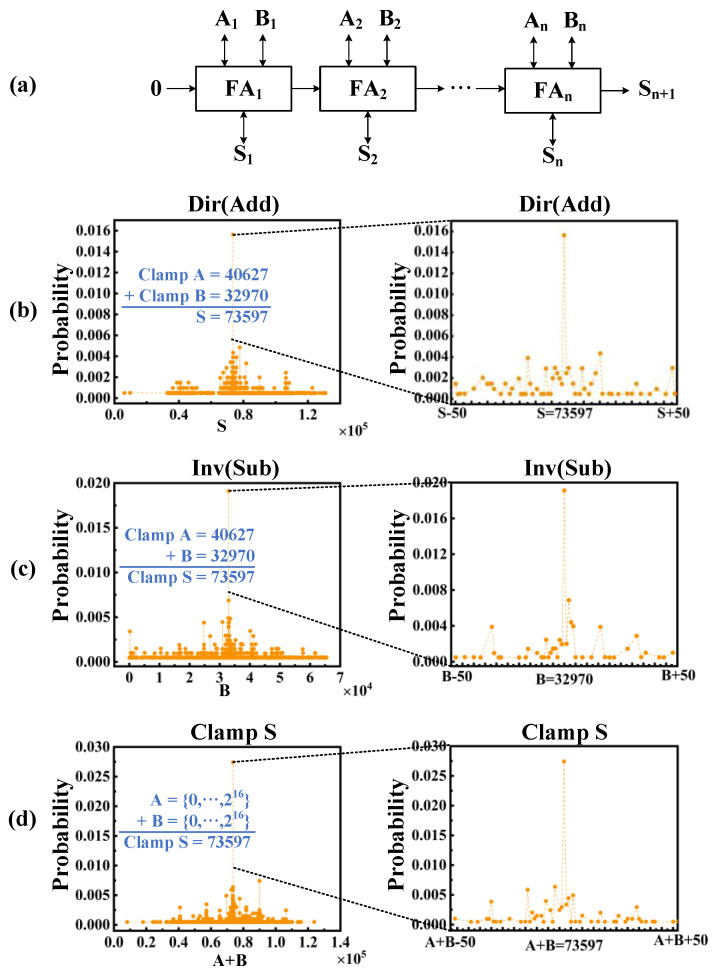
Invertible 16-bit RAC operation. (**a**) N-bit RCA is designed based on constructed invertible full adders; (**b**) clamping A and B working in directional mode (used for addition). The left is the statistics of S over a period, and the right is the statistics of 100 samples around the correct S; (**c**) clamping A and S working in inverse mode (used for subtraction); (**d**) only clamping S, and the statistics of A + B.

We cascade the constructed invertible FAs to form an N-bit RCA as shown in Figure 8a, and randomly select the data *A* = 40,627, *B* = 32,970 as an example to illustrate the three different modes of the invertible 16-bit RCA. In Figure 8b, RCA works in the directional mode (used for addition), *A* and *B* are clamped through *Clamp* and *Fix_n_* signals, and *S* is countered for a long time. The probability of the correct result is significantly high (near 0.016) among the 2^17^ possibilities. In Figure 8c, we clamp *A* and *S* to use RCA as a subtraction in inverse mode, and the probability of *B* = 32,970 is much higher than the others, which are close to 0.019. In Figure 8d, we clamp only *S* and calculate the sum of *A* and *B*. The highest probability (close to 0.028) is for *A* + *B* = 73,597. Figure 8d shows a great ability to extract the correct answer despite large fluctuations, which is useful in some NP problems such as the Subset Sum Problem. The invertible 16-bit RCA requires 80 clock cycles to complete a calculation.

### 4.3. 4-Bit Multiplier

**Figure 9:**
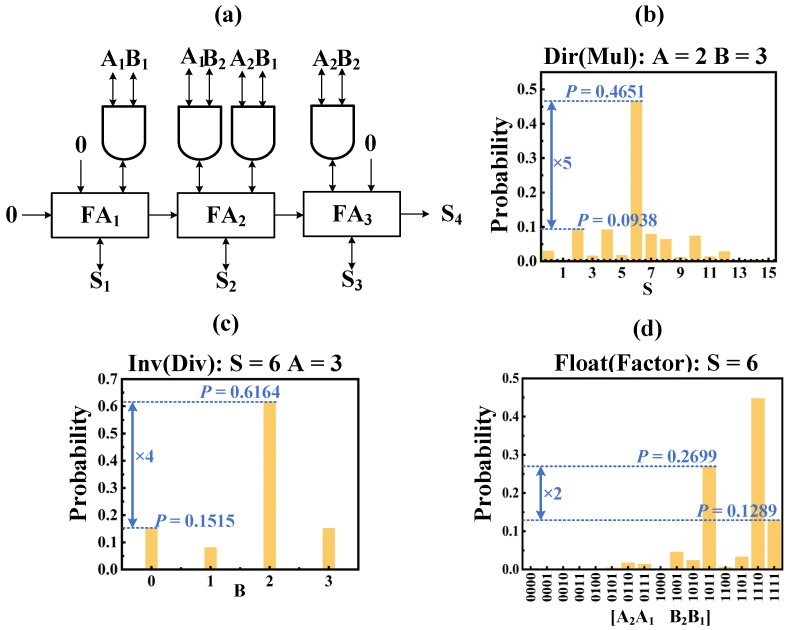
Invertible 4-bit multiplier operation. (**a**) The structure of 4-bit multiplier based on invertible AND gates and FA; (**b**) multiplication: Clamping A = 2, B = 3, the statistics of S; (**c**) division: Clamping A = 2, S = 6, the statistics of B; (**d**) factorization: Clamping S = 6, the statistics of A and B.

We construct a 4-bit multiplier consisting of four invertible AND gates and three invertible FAs as shown in Figure 9, which can be applied to multiplication, division, and factorization. We take 2 × 3 = 6 as an example to illustrate these three functions of the multiplier. In the multiplication process, we clamp A = 2, B = 3, and count S ([S4, S3, S2, S1]), and S = 6 appears with the highest probability, close to 0.45. In this division, we clamp A = 2, S = 6, and count B ([B2, B1]), with B = 2 taking place with the highest probability, close to 0.6. In factorization, we just clamp S = 6 and count A and B ([A2, A1, B2, B1]), and the relatively high probability of (1101) and (1011) implies that A = 2, B = 3 or A = 3, B = 2 is more likely. An invertible 4-bit multiplier requires 18 clock cycles to complete a calculation.

## 5. Conclusions

In this work, invertible logic circuits are realized by combining RRAM and digital circuits. Table 2 shows a comparison between the previous work and this work in terms of a hardware implementation of invertible logic. We design an RRAM-based TRNG to replace the pseudo-random number generator formed by the linear feedback shift register (LFSR) and combined it with the activation function implemented by PWL to form a standard p-bit unit. We also propose a p-bits multiplexing strategy to save hardware resources. Since our work is primarily based on a digital circuit approach, we also compare this work with two previous works on purely digital circuit implementations in terms of hardware resources such as the number of LUTs and registers required, as shown in Table 3. Table 3 shows the number of LUTs and registers used in this work, [4], and [5] to implement invertible AND gates, invertible FA, and invertible 32-bit RCA, respectively. The percentage based on [5] can more intuitively illustrate the superiority of this work in saving resource utilization. We use the Xilinx Kintex ultrascale XCKU0401-FBVA676 FPGA (San Jose, CA, USA). From the circuit structure analysis, the RRAM-based TRNG uses more than 100 times fewer transistors than the LFSR [9], and our method has greatly reduced hardware resources.

## Figures and Tables

**Table 1:** The truth table of the 15-segment polyline approximates the sigmoid function for x > 0.

Input Range	Input Value	Output Range	Output Value	Slope
[0, 1)	0000xxxxxxxxxxxx	[0.5, 0.75)	10xxxxxxxxxxxxxx	0.25
[1, 2)	0001xxxxxxxxxxxx	[0.75, 0.875)	110xxxxxxxxxxxxx	0.125
[2, 3)	0010xxxxxxxxxxxx	[0.875, 0.9375)	1110xxxxxxxxxxxx	0.0625
[3, 4)	0011xxxxxxxxxxxx	[0.9375, 0.96875)	11110xxxxxxxxxxx	0.03125
[4, 5)	0100xxxxxxxxxxxx	[0.96875, 0.984375)	111110xxxxxxxxxx	0.015625
[5, 6)	0101xxxxxxxxxxxx	[0.984375, 0.9921875)	1111110xxxxxxxxx	0.0078125
[6, 7)	0110xxxxxxxxxxxx	[0.9921875, 0.99609375)	11111110xxxxxxxx	0.00390625
[7, 8)	0111xxxxxxxxxxxx	[0.99609375, 1)	11111111xxxxxxxx	0.001953125

**Table 2:** Hardware implements of invertible logic.

Ref.	P-Bit		Output Value
	RNG	Activation Function	
[3]	MCU	MCU (sigmoid)	MCU
[4]	LFSR	LUT (tanh)	FPGA
[5]	LFSR	FSM (tanh)	ASIC and FPGA
[6]	MTJ	No need	Resistance network
This work	RRAM	PWL (sigmoid)	FPGA

**Table 3:** FPGA resource utilization of the invertible logic circuit.

Invertible Logic Circuit	This Work		[5]		[4]		This Work/[5]	
	LUTs	Registers	LUTs	Registers	LUTs	Registers	LUTs	Registers
AND	45	59	257	307	156	123	17.5%	19.2%
FA	85	76	400	329	1345	586	21.3%	23.1%
32-bit RCA	2731	1678	10,455	1910	38,814	18,071	26.1%	87.9%

## Data Availability

The data that support the findings of this study are available from the corresponding author upon request.
